# Decreased Serum Maresin 1 Concentration Is Associated With Postmenopausal Osteoporosis: A Cross-Sectional Study

**DOI:** 10.3389/fmed.2021.759825

**Published:** 2022-01-10

**Authors:** Jing Wu, Xin-Yue Li, Xia Fang, Fang-Yuan Teng, Yong Xu

**Affiliations:** ^1^Department of Endocrinology and Metabolism, The Affiliated Hospital of Southwest Medical University, Luzhou, China; ^2^Cardiovascular and Metabolic Diseases Key Laboratory of Luzhou, The Affiliated Hospital of Southwest Medical University, Luzhou, China; ^3^Sichuan Kidney Disease Clinical Medicine Research Center, The Affiliated Hospital of Southwest Medical University, Luzhou, China; ^4^Metabolic Vascular Disease Key Laboratory of Sichuan Province, The Affiliated Hospital of Southwest Medical University, Luzhou, China; ^5^Experimental Medicine Center, The Affiliated Hospital of Southwest Medical University, Luzhou, China

**Keywords:** postmenopausal osteoporosis (PMOP), bone mineral density (BMD), bone metabolism, Maresin 1, cross-sectional study

## Abstract

**Background:** Maresin 1 plays a role in the regulation of inflammation and metabolic diseases *in vivo*. An increasing number of studies have reported that postmenopausal osteoporosis (PMOP) is associated with inflammation. However, the potential relationship between the serum Maresin 1 content and PMOP is unclear.

**Aims:** 1) To evaluate the Maresin 1 content in postmenopausal women with osteopenia, osteoporosis, or without these conditions (normal group) and 2) to analyze the correlations between Maresin 1 concentrations and bone mineral density (BMD) and bone turnover markers.

**Methods:** In this cross-sectional study, we measured serum Maresin 1 concentrations, serum biochemical parameters, markers of bone metabolism, and BMD of the femoral neck, lumbar spine, and hip in 141 postmenopausal women.

**Results:** We found that serum Maresin 1 in the osteopenia (140.09 ± 30.54 pg/ml) and PMOP (124.68 ± 31.35 pg/ml) groups were significantly lower than those in the normal group (167.38 ± 24.85 pg/ml) (*P* < 0.05 and *P* < 0.001). Serum Maresin 1 levels were positively correlated with femoral neck, lumbar spine, and hip BMD (*P* < 0.001). Meanwhile, Maresin 1 concentrations were positively associated with 25-hydroxyvitamin D [25(OH)D] levels (*P* < 0.001), but negatively correlated with β-CrossLaps of type 1 collagen containing cross-linked C-telopeptide (β-CTX) (*P* = 0.002), procollagen type I amino-terminal propeptide (PINP) (*P* = 0.004), tartrate-resistant acid phosphatase 5b (TRAP-5b) (*P* = 0.005), and osteocalcin (OC) levels (*P* = 0.001). Multivariate logistic regression analysis showed that a decrease in Maresin 1 concentration was still associated with osteopenia (*P* = 0.035) or PMOP (*P* = 0.016). Maresin 1 levels had a maximum area under curve of 0.820 for osteopenia and 0.746 for PMOP (*P* < 0.001). Our results showed that the serum Maresin 1 levels were reduced in osteopenia and PMOP patients compared with that in normal subjects, and were the lowest in the PMOP subjects. The results suggest that Maresin 1 may serve as a new non-invasive diagnostic biomarker for PMOP.

## Introduction

Osteoporosis (OP) is a metabolic skeletal disorder characterized by decreased bone mineral density (BMD) and an increased risk of fracture due to abnormal bone microstructure (1). It is usually characterized by excruciating pain and an increased risk of fracture. According to a recent epidemiological study, OP is the cause of ~1,000 fractures per hour worldwide and ~9 million fractures annually (2). OP results in a decreased quality of life and an increased incidence of disability and mortality, which is associated with an increased economic burden on society (3, 4). Postmenopausal osteoporosis (PMOP) is the most common type of primary OP (5). The incidence of osteoporosis in women over 60 is nearly 50%, and with the aging of the global population, the incidence of PMOP is expected to increase rapidly. As a result, the bone health of postmenopausal women deserves close attention (6, 7).

As a chronic disease, there is a need to search for and identify further potential biochemical markers for PMOP. Protective biomarkers play an important role in predicting disease occurrence, prognosis, and treatment, and can improve safety and reduce economic costs compared with imaging examinations. However, there is currently a lack of research on protective biomarkers of PMOP. Therefore, the identification of potential novel protective biomarkers of PMOP is needed. Recent studies have demonstrated that inflammation can disrupt bone metabolism and increase bone loss, which is an important cause of PMOP (8). It has been shown that pro-inflammatory factors, such as interleukin-1 (IL-1), interleukin-6 (IL-6), and tumor necrosis factor-α (TNF-α), play important roles in PMOP (9). Estrogen deficiency can lead to the activation of nuclear factor κB (NF-κB) signaling in osteoclasts, thereby increasing bone resorption (10).

Maresin 1 is a lipid molecule synthesized in macrophages by an omega-3 polyunsaturated fatty acid, docosahexaenoic acid (DHA), and has been identified as a new type of inflammatory mediator (11). Multiple studies have demonstrated that Maresin 1 can control the inflammatory response by inhibiting neutrophil infiltration, downregulating the production of pro-inflammatory mediators, inhibiting the activation of NF-κB, improving Treg/Th17 imbalance, and alleviating endoplasmic reticulum stress (12–16). Notably, studies have also recently revealed that Maresin 1 plays an important role in obesity (17), non-alcoholic fatty liver disease (18), and type 2 diabetes (19), suggesting a significant relationship between Maresin 1 and metabolic disease.

In addition, studies have suggested that eating foods rich in DHA can improve bone metabolism, increase bone mass, and reduce the risk of PMOP (20, 21). It is not clear whether Maresin 1, a metabolite of DHA, can affect bone metabolism. In recent years, Maresin 1 has been demonstrated to affect bone metabolism. Recent studies have shown that Maresin 1 can increase osteoblast differentiation by changing the macrophage phenotype (19, 22). In addition, because Maresin 1 has a strong anti-inflammatory effect and can inhibit the expression of NF-κB, it may affect RANKL-RANK signaling and downregulate osteoclastogenesis (23–25). This indicates that there is a potential relationship between Maresin 1 and PMOP, which requires further study.

At present, studies on the potential relationship between serum Maresin 1 levels and PMOP disease progression are lacking. To address this, we investigated the expression of Maresin 1 in the serum of postmenopausal patients with non-osteoporosis, low bone mass, and osteoporosis to explore the correlation between Maresin 1 levels and the disease severity of PMOP in this study.

## Materials and Methods

### Study Subjects

Postmenopausal women aged 50–70 years who had been postmenopausal for at least 3 years were included in our study. A total of 141 postmenopausal women were enrolled, including 33 healthy subjects (NC), 48 osteopenia patients, and 60 osteoporosis patients (PMOP). OP was defined by BMD levels according to the World Health Organization (WHO) diagnostic criteria (26). The subjects were grouped according to their T-score: NC (T-score > −1), osteopenia (−2.5 > T-score > −1), and PMOP (T-score < −2.5).

The exclusion criteria were as follows: (1) suffering from chronic diseases, including diabetes, hypertension, hyperparathyroidism, and gastritis; (2) a history of smoking and drinking; (3) after hysterectomy and oophorectomy; and (4) women who received any drugs affecting bone metabolism in the last 6 months, including antibiotics, cortisol-like hormones, and anti-OP treatment.

The study was approved by the Ethics Committee of the Affiliated Hospital of Southwest Medical University and registered online (Clinical Trial Register No. ChiCTR2100045168) according to the Declaration of Helsinki. All subjects provided written informed consent prior to participation.

### Clinical Parameters

The subjects completed a written health questionnaire at the start of the study, including information on their age, menopausal status, medical history, smoking history, history of alcohol use, drug use status in the past 6 months, and the occurrence of radiographically confirmed low traumatic fracture (27). The subjects' height, weight, waist circumference, and fasting plasma glucose (FPG) were measured, and their body mass index (BMI) was calculated based on the height and weight (kg/m^2^). Weight (kg) was measured to the nearest 0.1 kg, and height and waist (cm) were measured to the nearest 0.1 cm.

### Laboratory Tests

Blood samples were collected between 8:00 and 10:00 a.m. after an overnight fast (>8 h). These were then centrifuged for 20 min (2,000–3,000 rpm), and the supernatant was carefully collected (28). Total cholesterol (TC), triglyceride (TG), high-density lipoprotein cholesterol (HDL-C), low-density lipoprotein cholesterol (LDL-c), Ca, and P were detected using a biochemical autoanalyzer (Beckman CX-7 Biochemical Autoanalyzer; Beckman Coulter, Brea, CA, USA), and the 25(OH)D levels were assessed by DiaSorin radioimmunoassay (DiaSorin Inc., Stillwater, MN, USA). Intact parathyroid hormone (PTH) and β-CrossLaps of type 1 collagen containing cross-linked C-telopeptide (β-CTX), procollagen type 1 N-terminal propeptide (P1NP), and osteocalcin (OC) were measured using an automated Roche electrochemiluminescence system (Roche Diagnostic GmbH, Mannheim, Germany). Tartrate-resistant acid phosphatase 5b (TRAP-5b) was measured using the tartrate resistant acid phosphatase assay kit (Beyotime, Shanghai, China).

### Assessment of Serum Maresin 1 Levels

The serum Maresin 1 content was determined by ELISA (Maresin 1 ELISA kit; Cayman Chemical, Ann Arbor, MI, USA). The intra-and inter-assay variations in serum Maresin 1 evaluation were 4–6% and 6–8%, respectively. Each sample was tested three times, and the results were averaged.

### BMD Measurements

The BMD (g/cm^2^) of the left femoral neck (FN), lumbar vertebra 1–4 (L1-L4), and total hip were measured using DXA (Hologic QDR 4500; Hologic Inc., Waltham, MA, USA). The lunar device was calibrated daily. All scans and analyses were performed by the same technician throughout the study.

### Statistical Analysis

For continuous variables, the normal distribution data were expressed as the mean ± standard deviation (SD), while the non-normal distribution data were described as the median and quaternary ranges. The Kolmogorov-Smirnov test was used to check for normal distribution. Several variables were represented as a skewness distribution and were placed into a normal distribution through a logarithmic transformation of the data before statistical analysis. One-way ANOVA with Tukey's *post-hoc* test was performed for multiple comparisons. The Pearson correlation coefficient was used to analyze the correlations between the serum Maresin 1 levels and the bone metabolism indices or BMD. Multivariate logistic regression analyses were used to analyze the relationship between the serum Maresin 1 concentration and osteopenia and PMOP. Receiver operating characteristic (ROC) curve analysis was used to determine the area under the curve (AUC), diagnostic sensitivity, and specificity of Maresin 1. All statistical analyses were performed using Stata 23.0 (StataCorp LP, College Station, Texas, USA). A two-sided *P* < 0.05, was considered statistically significant.

## Results

### Baseline Characteristics

The clinical and laboratory characteristics of the subjects are presented in Table 1. Age, BMI, waist circumference, FPG, TC, TG, HDL-c, and LDL-c levels were not significantly different among the three groups (*P* > 0.05).

**Table 1:** Clinical and laboratory characteristics of the study subjects.

**Parameter**	**NC**	**Osteopenia**	**PMOP**
No. of subjects	33	48	60
Age (years)	56.3 (52–58.5)	57.4 (56–59)	57.72 (55–60)
BMI (kg/m^2^)	23.19 (20.76–25.26)	23.73 (21.07–25.37)	22.93 (20.64–25.34)
Waist (cm)	79.85 ± 9.98	81.33 ± 7.81	79.30 ± 9.34
FPG (mmol/L)	4.94 ± 0.35	4.94 ± 0.43	4.98 ± 0.39
**Serum**			
TC (mmol/L)	5.00 ± 0.81	5.26 ± 0.95	5.13 ± 1.03
TG (mmol/L)	1.10 (0.84–1.50)	1.29 (0.99–1.73)	1.24 (0.91–2.03)
HDL-C (mmol/L)	1.52 ± 0.41	1.50 ± 0.32	1.56 ± 0.35
LDL-C (mmol/L)	3.20 ± 0.79	3.32 ± 0.96	3.06 ± 0.80
Ca (mmol/L)	2.38 ± 0.09	2.34 ± 0.07^a^	2.34 ± 0.07^b^
P (mmol/L)	1.26 ± 0.17	1.30 ± 0.14	1.30 ± 0.13
PTH (pg/ml)	28.63 (26.64–31.20)	30.21 (28.66–32.43) ^a^	31.02 (29.32–33.73)^b^
25(OH)D (ng/ml)	31.34 ± 3.67	31.15 ± 2.82	29.14 ± 3.52^b^^d^
β-CTX (ng/ml)	0.43 ± 0.08	0.48 ± 0.07^b^	0.51 ± 0.08^b^
PINP (ng/ml)	42.87 ± 4.60	44.67 ± 3.71	51.79 ± 4.34^b^^d^
TRAP-5b (U/L)	4.26 ± 0.31	4.87 ± 0.44^b^	4.88 ± 0.43^b^
OC (ng/ml)	15.06 ± 1.95	15.22 ± 1.94	16.08 ± 1.86^a^^c^
Maresin 1 (pg/ml)	167.38 ± 24.85	140.09 ± 30.54^b^	124.68 ± 31.35^b^^d^
**BMD (g/cm^2^)**			
FN	0.93 ± 0.05	0.83 ± 0.05 ^b^	0.75 ± 0.05^b^^d^
L1-4	1.15 ± 0.08	0.98 ± 0.09^b^	0.87 ± 0.10^b^^d^
Hip	0.98 ± 0.07	0.88 ± 0.08^b^	0.78 ± 0.10^b^^d^

*Normally distributed data are presented as the mean ± SD, data that are not normally distributed data are shown as the median (interquartile range)*.

NC, normal; PMOP, postmenopausal osteoporosis; BMI, body mass index; FPG, fasting plasma glucose; TC, total cholesterol; TG, triglyceride; HDL-c, high-density lipoprotein-cholesterol; LDL-c, low-density lipoprotein-cholesterol; PTH, parathyroid hormone; 25(OH)D, 25-hydroxyvitamin D; β-CTX, β-CrossLaps of type 1 collagen containing cross-linked C-telopeptide; PINP, procollagen type 1 N-terminal propeptide; TRAP-5b, tartrate-resistant acid phosphatase 5b; OC, osteocalcin; BMD, bone mineral density; FN, femoral neck; L1-4, lumbar vertebra 1-4.

a *P < 0.05 compared with Normal*.

b *P < 0.01 compared with Normal*.

c *P < 0.05 compared with Osteopenia*.

d *P < 0.01 compared with Osteopenia*.

In terms of the bone metabolism parameters, Ca was significantly higher in osteopenia and PMOP patients than in NC subjects (*P* < 0.05 and *P* < 0.01, respectively). The serum P-levels were not significantly different among the three groups (*P* > 0.05). The PTH levels were significantly higher in osteopenia and PMOP patients than in NC subjects (*P* < 0.05, *P* < 0.01, respectively). 25(OH)D was significantly reduced in PMOP patients compared with NC (*P* < 0.01) and osteopenia patients (*P* < 0.01), while no significant difference was found between the osteopenia and NC groups (*P* > 0.05). β-CTX was significantly higher in the osteopenia and PMOP patients than in the NC subjects, and was the highest in PMOP (*P* < 0.01). Meanwhile, PINP was significantly higher in patients with PMOP than in those with osteopenia (*P* < 0.01) and NC subjects (*P* < 0.01). The levels of TRAP-5b were significantly higher in the osteopenia and PMOP patients than in the NC subjects (*P* < 0.01). OC was significantly higher in patients with PMOP than in those with osteopenia (*P* < 0.05) and the NC subjects (*P* < 0.05).

As for the BMD parameters, femoral neck, lumbar vertebra 1–4, and hip BMD were significantly reduced in the osteopenia and PMOP patients compared with the NC subjects, and were the lowest in PMOP (*P* < 0.01).

### Comparison of Serum Maresin 1 Content Between Different Groups

**Figure 1:**
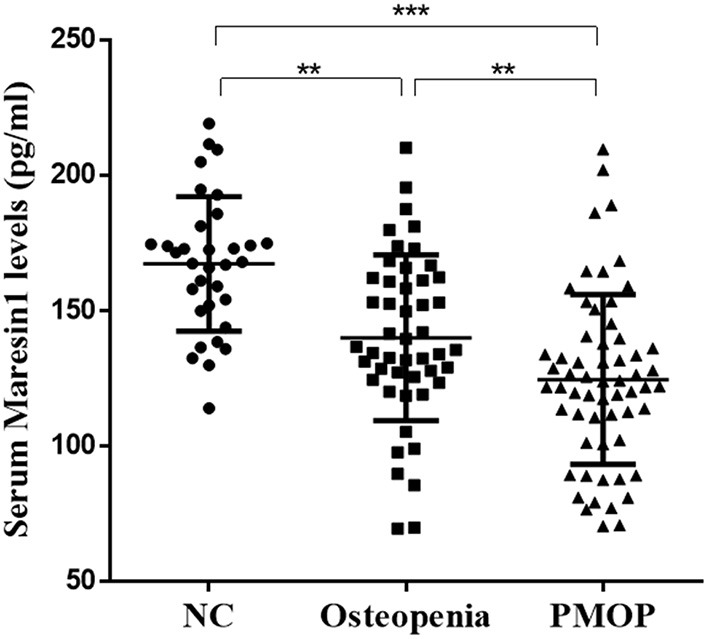
Scatter plots of serum Maresin 1 concentration of subjects with different BMD. Each data point represents a serum sample, with the median line of the horizontal line for each dataset representing the mean, and the boundaries of the vertical line representing the SD. ***P < 0.001 compared with NC; **P < 0.05 compared with osteopenia.

As shown in Figure 1, the serum Maresin 1 levels were significantly decreased in the osteopenia (140.09 ± 30.54 pg/ml) and PMOP (124.68 ± 31.35 pg/ml) subjects compared to the NC subjects (167.38 ± 24.85 pg/ml) (*P* < 0.05 and *P* < 0.001). The level of serum Maresin 1 in the PMOP group was lower than that in the osteopenia group (*P* < 0.05).

### Association of Serum Maresin 1 Levels With Bone Turnover Marker and BMD

**Figure 2:**
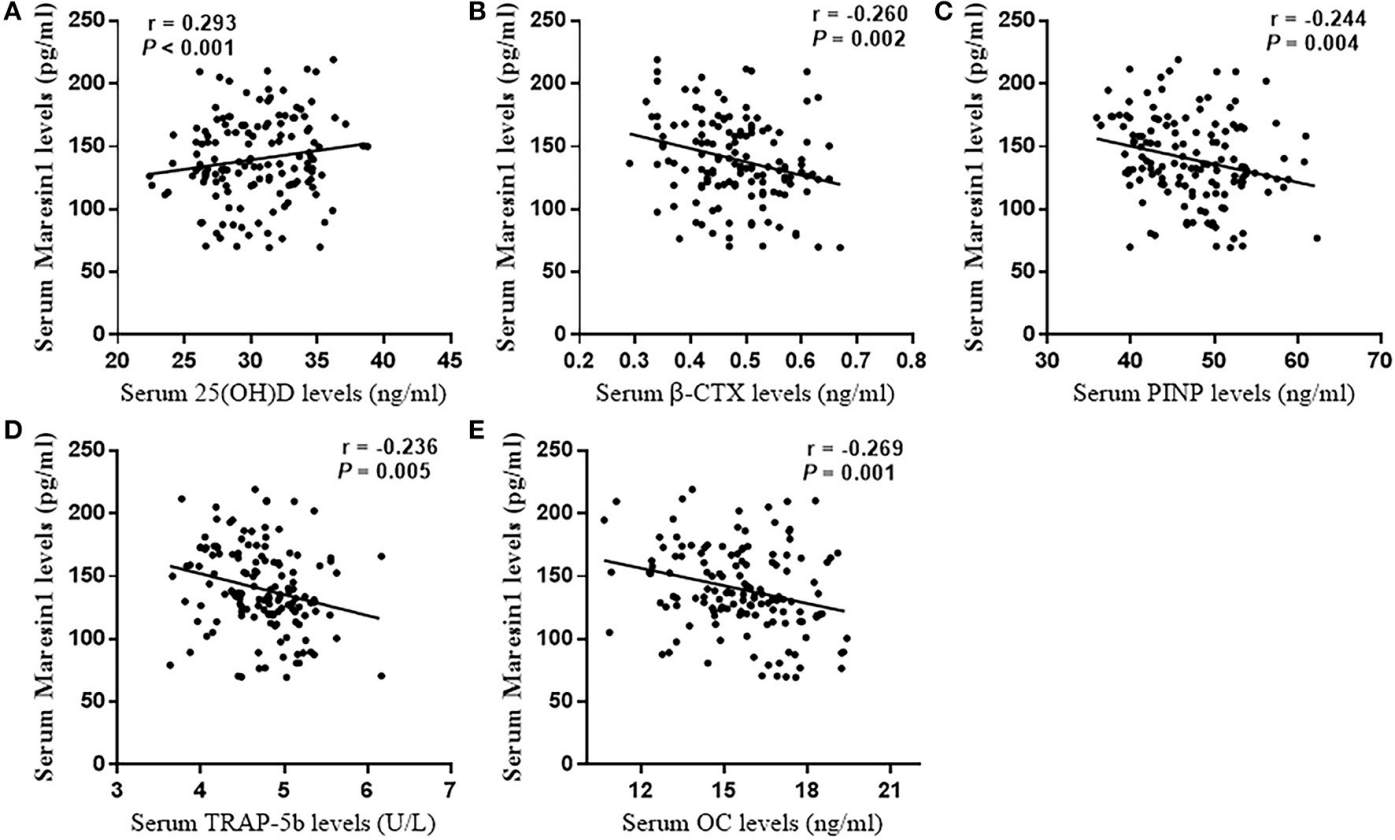
Correlation of Maresin 1 concentration with bone metabolism. **(A)** Association of serum Maresin 1 levels with serum 25(OH)D levels. **(B)** Association of serum Maresin 1 levels with β-CTX serum levels. **(C)** Association of serum Maresin 1 levels with serum PINP levels. **(D)** Association of serum Maresin 1 levels with serum TRAP-5b levels. **(E)** Association of serum Maresin 1 levels with serum OC levels.

**Figure 3:**
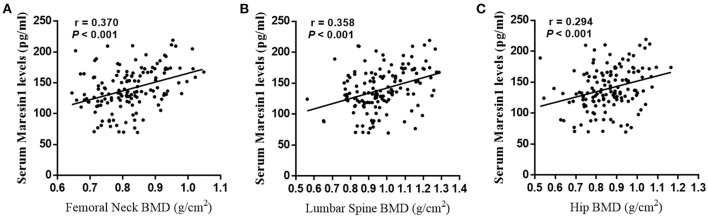
Correlation of Maresin 1 concentration with BMD. **(A)** Association of serum Maresin 1 levels with femoral neck BMD. **(B)** Association of serum Maresin 1 levels with lumbar spine BMD. **(C)** Association of serum Maresin 1 levels with hip BMD.

The **s**erum Maresin 1 levels were positively correlated with the serum 25(OH)D concentration (*r* = 0.293, *P* < 0.001; Figure 2A) and negatively correlated with the serum β-CTX concentration (*r* = −0.260, *P* = 0.002; Figure 2B), PINP (*r* = −0.244, *P* = 0.004; Figure 2C), TRAP-5b (*r* = −0.236, *P* = 0.005; Figure 2D), and OC (*r* = −0.269, *P* = 0.001; Figure 2E). The relationship between the serum Maresin 1 levels and BMD was also investigated. We found that the serum Maresin 1 levels were positively correlated with BMD at the femoral neck (*r* = 0.370, *P* < 0.001; Figure 3A), lumbar spine (*r* = 0.358, *P* < 0.001; Figure 3B), and hip (*r* = 0.294, *P* < 0.001; Figure 3C). These findings indicate that the Maresin 1 concentration is strongly related to markers of bone metabolism and BMD.

Furthermore, as shown in Table 2, after controlling for age, BMI, waist circumference, FPG, lipid profiles, Ca, P, PTH, 25(OH)D, β-CTX, PINP, TRAP-5b, and OC, multivariate logistic regression analysis revealed that decreased serum Maresin 1 levels were significantly associated with osteopenia (OR = 0.807, 95% CI 0.660–0.985, *P* = 0.035) and PMOP (OR = 0.781, 95% CI 0.638–0.955, *P* = 0.016).

**Table 2:** Multivariate logistic regression analysis with serum Maresin 1 levels^a^.

	**Serum Maresin 1 (pg/ml)**		
	**Odds ratio (OR)**	**95% CI**	***P-value***
Osteopenia	0.807	0.660–0.985	0.035
PMOP	0.781	0.638–0.955	0.016

*CI, confidence interval; PMOP, postmenopausal osteoporosis*.

a *Main model is adjusted for age, BMI, Waist, FPG, TC, TG, HDL-C, LDL-C, Ca, P, PTH, 25(OH)D, β-CTX, PINP, TRAP-5b, OC*.

### Receiver Operating Characteristic Curve Analysis

**Figure 4:**
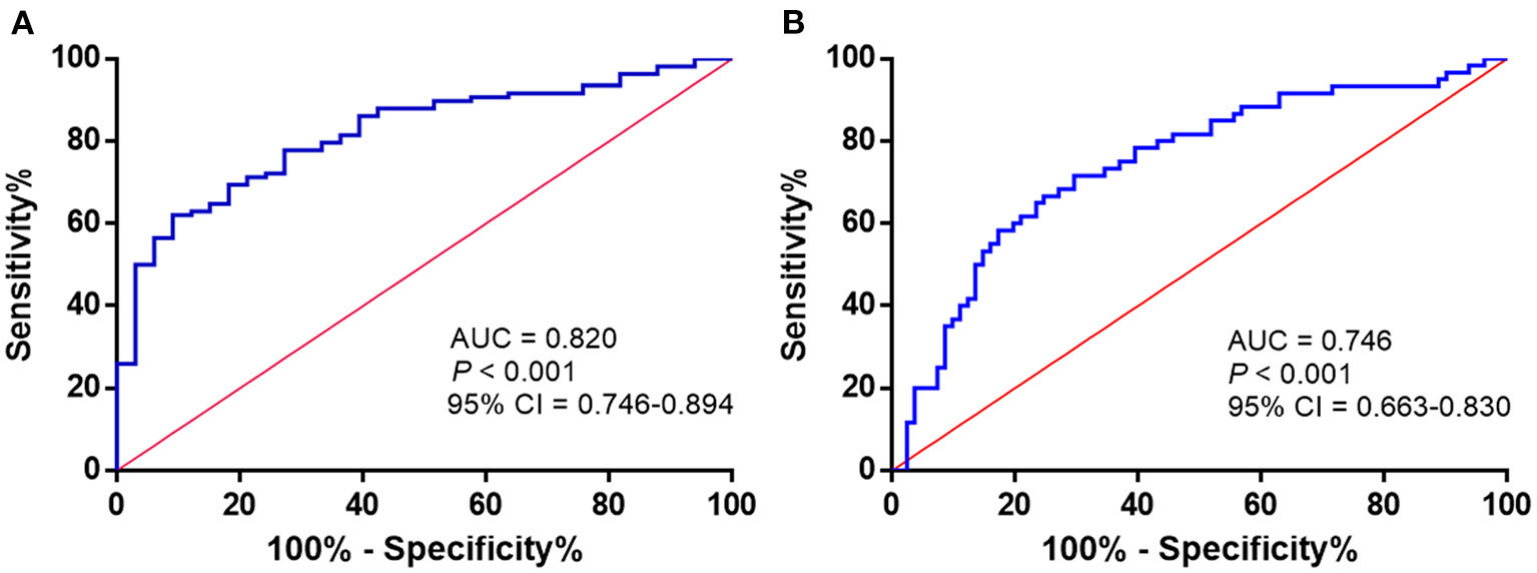
ROC curve analysis to determine serum Maresin 1 levels (pg/mL) in osteopenia **(A)** and PMOP **(B)**.

To assess the potential diagnostic value of Maresin 1 for osteopenia and PMOP, ROC analysis was conducted, and the associated AUC was used to confirm the diagnostic value of Maresin 1. The AUC of Maresin 1 for osteopenia was 0.820 (95% CI 0.746–0.894, *P* < 0.001; Figure 4A), and 0.746 for PMOP (95% CI 0.663–0.830, *P* < 0.001; Figure 4B). ROC analysis determined a cut-off value for the serum Maresin 1 levels of >158.7 pg/mL between the normal and osteopenia subjects, and of >134.0 pg/mL between the normal and PMOP subjects. The sensitivity and specificity evaluated with the optimal cutoff points are shown in Table 3.

**Table 3:** Sensitivity and specificity evaluated with optimal cutoff points.

	**Serum Maresin 1 (pg/ml)**		
	**Sensitivity (%)**	**Specificity (%)**	**Criterion**
Osteopenia	79.63	66.67	<158.7
PMOP	71.67	70.37	<134.0

*PMOP, postmenopausal osteoporosis*.

## Discussion

To our knowledge, this is the first population-based study to examine the association of serum Maresin 1 levels with markers of bone metabolism and BMD in postmenopausal women. In our study, the serum Maresin 1 levels were found to be significantly lower in osteopenia and PMOP patients than in healthy subjects. In addition, these PMOP patients showed the lowest Maresin 1 levels among the three groups. In addition, we also observed that the serum Maresin 1 levels were significantly correlated with disease severity, as determined by markers of bone metabolism and BMD. Multivariate logistic regression analysis showed that a decrease in the serum Maresin 1 levels was significantly associated with osteopenia or PMOP. The Maresin 1 levels had a maximum AUC of 0.820 for osteopenia and 0.746 for PMOP. Further ROC analysis indicated that decreased serum Maresin 1 levels could be used as a diagnostic indicator for osteopenia or PMOP. These findings suggested that Maresin 1 may act as a protective factor against PMOP.

Maresins are newly discovered macrophage inflammatory mediators, wherein Maresin 1 is a newly identified mediator with strong anti-inflammatory and pro-inflammatory regression activities (11). Maresin 1 can limit polymorphonuclear neutrophil (PMN) infiltration, stimulate macrophage uptake, and promote the clearance of apoptotic cells (29). A previous study (30) reported that Maresin 1 reduced the total number of leukocytes and PMNs in acute peritonitis. Recently, Tian Miao et al. found that the Maresin 1 levels were not significantly correlated with hsCRP levels in diabetic patients (19). Previous *in vitro* and *in vivo* studies have shown that Maresin 1 improves insulin sensitivity in diet-induced obese mice (17), delays the progression of non-alcoholic fatty liver disease (18), plays a protective role in a mouse model of colitis (31), and ameliorates Treg/Th17 imbalance in rheumatoid arthritis (32).

In recent years, the importance of inflammation in the regulation of bone metabolism has been extensively studied. Human and animal studies have shown that proinflammatory cytokines can promote bone resorption. A previous study showed that macrophages have different functional phenotypes, with pro-inflammatory M1 polarity and anti-inflammatory M2 polarity. M1 macrophages promote bone destruction by secreting pro-inflammatory factors, such as TNF-α, IL-6, and IL-1β, while M2 macrophages inhibit the activation of M1 macrophages by secreting anti-inflammatory factors, such as IL-10, thereby inhibiting bone resorption. In addition, Maresin 1 tilts macrophages toward the M2 phenotype and increases M2 macrophage biosynthesis (33). Among them, Mundy et al. recently reported that IL-1, IL-6, and TNF-α play an important role in estrogen-deficient bone loss (9). Recently, Hao et al. found that the serum Maresin 1 levels markedly decreased the TNF-α and IL-6 levels in septic mice (34). The lack of estrogen in postmenopausal women leads to the activation of NF-κB signaling in osteoclasts to increase bone resorption (10, 35), and the activation of the NF-κB signaling pathway in osteoblasts inhibits bone formation (36). Notably, Maresin 1 has been reported to inhibit NF-κB activation in many aspects (23–25). Qiu et al. recently reported that Maresin 1 decreased the nuclear displacement of NF-κB in a renal ischemia perfusion reinjury model (23). Maresin 1 alleviated concanavalin A-induced acute liver injury in mice by significantly inhibiting the NF-κB signaling pathway (24). Maresin 1 decreases inflammatory cytokine production and reduces nuclear NF-κB activation (25).

Recently, Miao et al. (19) indicated that Maresin 1 can alter the phenotype of macrophages, affecting macrophage-osteoblast crosstalk, which is a key component of bone regeneration, as well as regulating osteoblast differentiation and activity and promoting bone formation. Wang et al. (22) found that in a rat tooth extraction model, Maresin 1 acted as an anti-inflammatory and osteocyte proliferator that accelerated complete wound closure and stimulated faster fossa filling. Our results are consistent with these results and support the hypothesis that Maresin 1 is related to bone metabolism, with the ability to increase bone density.

Furthermore, Maresin 1 is known to be synthesized by DHA in macrophages (11). A previous study (20) reported that dietary supplementation rich in DHA can reduce the expression of inflammatory cytokines IL-6 and TNF-α, and significantly increase bone mineral density in elderly female mice. Moon et al. found that the T-score of the femoral neck was positively associated with DHA, and the risk of osteoporosis was negatively correlated with EPA + DHA in postmenopausal Korean women (21). These findings highlight the potential relationship between Maresin 1 and PMOP, however, further research is required to determine the precise functional mechanisms involved.

There were some limitations to this study. First, determining a causal relationship between the serum Maresin 1 levels and osteopenia or PMOP was difficult. Studies have found that Maresin 1 is an effective inflammatory mediator that has been shown to be associated with inflammation regression and tissue homeostasis, and these physiological functions are closely related to the occurrence and development of PMOP. Therefore, we conjecture that the decrease in serum Maresin 1 levels in osteopenia or PMOP patients may be due to a lack of inflammatory resolution. However, we cannot rule out that the decrease in the Maresin 1 levels may also be due to PMOP. Further prospective, longitudinal, and multicenter studies are required. Second, the present study was a cross-sectional study, wherein certain baseline characteristics were collected, including age, smoking history, history of alcohol use, and drug use history, among others, through questionnaires, which may lead to recall bias.

## Conclusion

The serum Maresin 1 levels were found to be significantly decreased in osteopenia or PMOP patients compared with healthy subjects, and were found to be the lowest in PMOP patients among these three groups. The Maresin 1 levels in the serum of postmenopausal women were closely related to several clinical parameters, including markers of bone metabolism and BMD. This study is the first to show that Maresin 1 may act as a protective biomarker for PMOP.

## Data Availability Statement

The original data in the study are included in the Supplementary Material, further inquiries can be directed to the corresponding authors.

## Ethics Statement

The studies involving human participants were reviewed and approved by the Ethics Committee of the Affiliated Hospital of Southwest Medical University. The patients/participants provided their written informed consent to participate in this study. Written informed consent was obtained from the individual(s) for the publication of any potentially identifiable images or data included in this article.

## Author Contributions

JW, X-YL, and XF: data collection and statistical analysis. JW and X-YL: writing the manuscript draft. F-YT and YX: revising manuscript content. JW, X-YL, XF, F-YT, and YX: assurance of data integrity. All authors contributed to manuscript drafting, study conception and design, review, and finalization.

## Funding

The work was supported by the project of Sichuan Kidney Disease Clinical Medicine Research Center (Grant No. 2019YFS0537-13), the project of Health Commission of Sichuan Province (Grant No. 21PJ090), the key projects of Sichuan Science and Technology Department (Grant Nos. 2019YFS0537 and 2020YFS0456), the grants from Luzhou-Southwest Medical University cooperation project (Grant No. 2018LZXNYD-PT01), and the Nation Natural Science Foundation of China (Grant No. 81970676).

## Conflict of Interest

The authors declare that the research was conducted in the absence of any commercial or financial relationships that could be construed as a potential conflict of interest.

## Publisher's Note

All claims expressed in this article are solely those of the authors and do not necessarily represent those of their affiliated organizations, or those of the publisher, the editors and the reviewers. Any product that may be evaluated in this article, or claim that may be made by its manufacturer, is not guaranteed or endorsed by the publisher.

## Abbreviations

OP: osteoporosis
BMD: bone mineral density
PMOP: postmenopausal osteoporosis
DHA: docosahexaenoic acid
FPG: fasting plasma glucose
TC: total cholesterol
TG: triglyceride
HDL-c: high-density lipoprotein-cholesterol
LDL-c: low-density lipoprotein-cholesterol
PTH: parathyroid hormone
25(OH)D: 25-hydroxyvitamin D
β-CTX: β-CrossLaps of type 1 collagen containing cross-linked C-telopeptide
PINP: procollagen type 1 N-terminal propeptide
TRAP-5b: tartrate-resistant acid phosphatase 5b
OC: osteocalcin
BMD: bone mineral density
FN: femoral neck
L1-4: lumbar vertebra 1-4
ROC: receiver operating characteristic
AUC: area under curve.
